# Finite deformation elastography of articular cartilage and biomaterials based on imaging and topology optimization

**DOI:** 10.1038/s41598-020-64723-9

**Published:** 2020-05-14

**Authors:** Luyao Cai, Eric A. Nauman, Claus B. W. Pedersen, Corey P. Neu

**Affiliations:** 1 Weldon School of Biomedical Engineering, Purdue University, West Lafayette, IN 47907 US; 2 School of Mechanical Engineering, Purdue University, West Lafayette, IN 47907 US; 3 Department of Basic Medical Sciences, Purdue University, West Lafayette, IN 47907 US; 4 Dassault Systèmes Deutschland GmbH, 20095 Hamburg, Germany; 5 Department of Mechanical Engineering, University of Colorado Boulder, Boulder, CO 80309 US

**Keywords:** Computational biophysics, Biomedical engineering

## Abstract

Tissues and engineered biomaterials exhibit exquisite local variation in stiffness that defines their function. Conventional elastography quantifies stiffness in soft (e.g. brain, liver) tissue, but robust quantification in stiff (e.g. musculoskeletal) tissues is challenging due to dissipation of high frequency shear waves. We describe new development of *finite deformation elastography* that utilizes magnetic resonance imaging of low frequency, physiological-level (large magnitude) displacements, coupled to an iterative topology optimization routine to investigate stiffness heterogeneity, including spatial gradients and inclusions. We reconstruct 2D and 3D stiffness distributions in bilayer agarose hydrogels and silicon materials that exhibit heterogeneous displacement/strain responses. We map stiffness in porcine and sheep articular cartilage deep within the bony articular joint space *in situ* for the first time. Elevated cartilage stiffness localized to the superficial zone is further related to collagen fiber compaction and loss of water content during cyclic loading, as assessed by independent *T*_2_ measurements. We additionally describe technical challenges needed to achieve *in vivo* elastography measurements. Our results introduce new functional imaging biomarkers, which can be assessed nondestructively, with clinical potential to diagnose and track progression of disease in early stages, including osteoarthritis or tissue degeneration.

## Introduction

The stiffness of a tissue, or its ability to resist deformation when subjected to an applied force, is associated with the structure of the extracellular matrix, a dynamic and biological aggregate of macromolecules that help to regulate the phenotype, expression, and differentiation of embedded cells. Abnormal stiffening or softening of tissue is often a functional hallmark of the pathologic, regenerative, or aging state in most organs in the body, including stiffening in liver fibrosis^1^ and developed tumors^2,3^, or softening in early development and invasive cancer cells^4,5^. The ability to capture the health and structure of a tissue, manifesting as microscale stiffness, represents a potential functional imaging biomarker with significant clinical utility for diagnosis of disease and repair.

The stiffness of tissues often changes during degeneration or pathology. In the articular cartilage lining the bony ends in our joints, softening and volumetric loss is a hallmark of osteoarthritis (OA), a degenerative joint disease that affects millions of people in the United States alone^6^ that often leads to pain, disability, and total joint arthroplasty. An unmet medical challenge is the diagnosis of early OA^7^, when emerging disease-rectifying therapies may be most effective^6,8^. However, conventional diagnostic methods for OA are largely insensitive to subtle morphological changes, and only reliably detect advanced OA^9^. Bulk softening of articular cartilage, attributed to the structural deterioration of the superficial collagen network, altered permeability, and depletion of proteoglycan content, has been linked to early degeneration in OA^10–13^. Local structural changes in the tissue, such as cartilage degradation differences through the (zonal) thickness, and among regional (e.g. anterior/posterior or load-/non-load-bearing) locations, present an opportunity to noninvasively probe tissue mechanical function and stiffness via imaging. Functional monitoring may be conducted spatiotemporally, especially between superficial and middle zones of cartilage, and may further assist in the design and *in situ* monitoring of engineered constructs post implantation^14^. Unfortunately, no conventional methods exist that enable noninvasive stiffness measurements of cartilage within the intact joint space.

**Figure 1:**
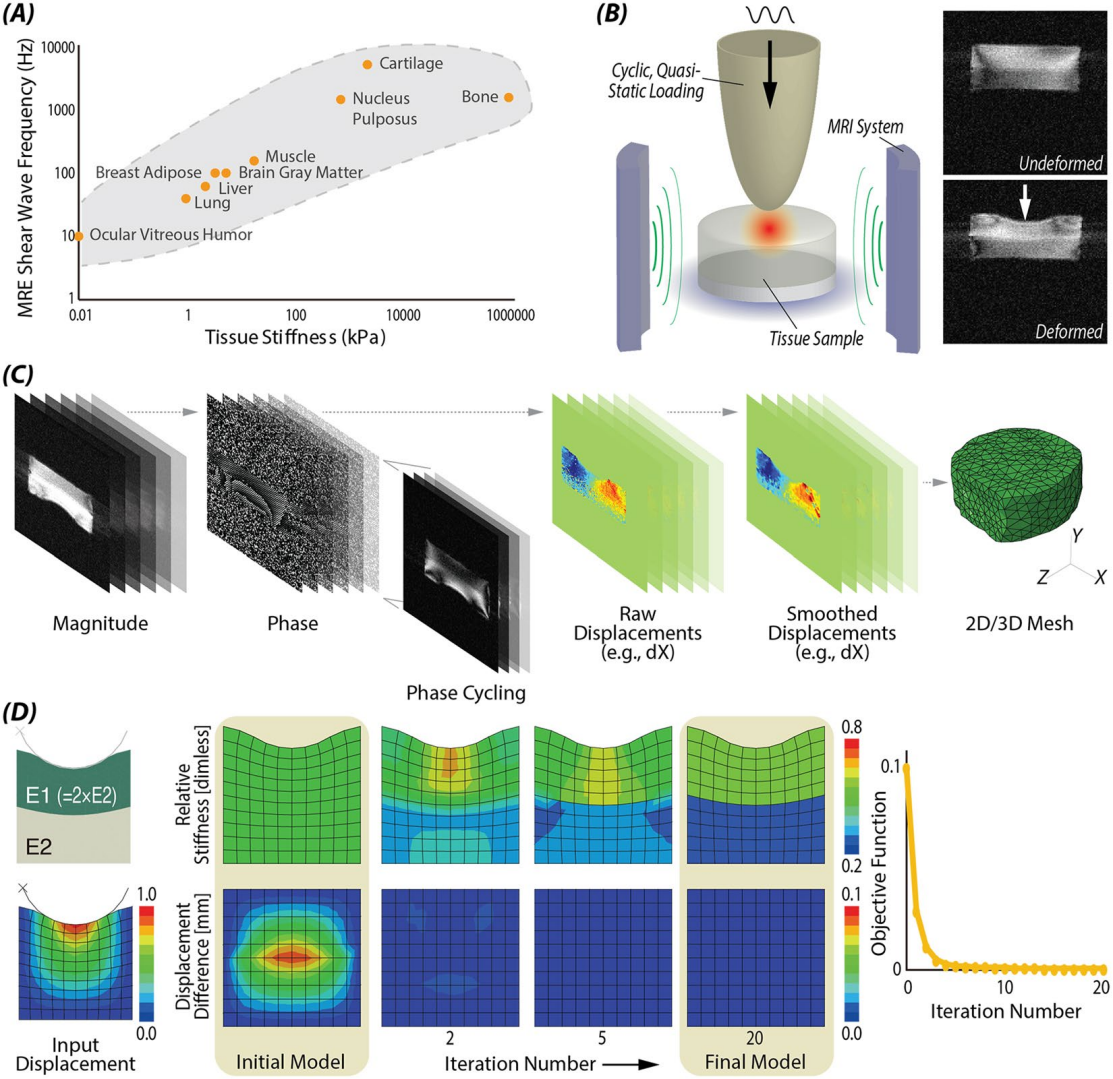
Finite deformation elastography workflow based on image acquisition and topology optimization. (**A**) Increased tissue stiffness demands high shear wave frequency in conventional MRE^57^. Instead, we use cyclic loading during MRI to enable large deformation imaging of stiff materials like cartilage. (**B**) Experimental setup of indentation test and undeformed and deformed morphology images; (**C**) dualMRI measured complex data from deformed tissue to extract phase maps that scale directly to displacements. Volume images were used to establish 2D and 3D mesh models. (**D**) Topology optimization was able to reconstruct a complex (e.g. bilayer) stiffness configurations by minimizing the difference of displacement between initial model and input (e.g. experimental) model.

Elastography provides a spatial map of mechanical quantities, including strain fields and elastic properties, using noninvasive (e.g. often imaging-based) tools to assess intra-tissue mechanics. Current elastography methods based on ultrasound, such as ultrasound elastomicroscopy^15^ or indentation^16^, and high-frequency ultrasound^17^, have demonstrated the ability to measure mechano-acoustic properties of *ex vivo* cartilage and revealed its relationship with tissue degradation. However, the weak scattering signals obtained from cartilage and the significant attenuation of the high-frequency ultrasound remains a challenge for imaging within intact joints^7^. Traditional magnetic resonance elastography (MRE), which is accomplished through synchronized imaging with shear wave excitation^18^, is able to map the mechanical properties of cartilage explants *in vitro*^19,20^. However, due to the large attenuation of high frequency waves required to probe stiff cartilage (Fig. 1), and limitations of current gradient systems to encode high frequency waves, it is still unclear whether shear wave-based MRE can be used to access cartilage deep within an intact joint or have sufficient spatial resolution to differentiate the thin cartilage (of 1~2 *mm* mean thickness) from the underlying subchondral bone of the articular joint^21^. In addition, unknown (force, displacement) boundary conditions of contacting tissues in the body further complicate measurements. For example, analysis of a separate musculoskeletal tissue, the nucleus pulposus in the intervertebral disc of the spine, demonstrated that shear properties were highly dependent on boundary and preload conditions, chosen frequency range, and wave signal-to-noise ratio (SNR)^22^. Similarly, articular cartilage contacts numerous complex (e.g. cartilage-cartilage, cartilage-meniscus, cartilage-bone) boundaries within the native joint space, variable stiffness and preload properties, and consequently reliable elastography strategies are unclear.

In order to directly measure displacement- and strain-based elastography in joint cartilage, we exploit a technique developed in our laboratory termed dualMRI (displacements under applied loading by MRI), which measures tissue deformation under exogenous mechanical loading^23,24^. Unlike high-frequency shear wave propagation in MRE, dualMRI utilizes physiological magnitudes of mechanical loading to mimic the walking cycle (e.g. compression magnitude and frequency) and ensure the mechanical stimuli propagate through the cartilage to closely mimic *in vivo* conditions. By acquisition and analysis of phase contrast data, displacements and strains can be computed with precision on the order of 11 μm and 0.1%, respectively, i.e. approaching the cellular scale^25^. dualMRI has been applied to investigate the strain pattern of human articular cartilage *in vivo*^26^, explanted human and bovine cartilage^25^, and intact joints and intervertebral discs^27^. Compared to shear wave based MRE, elastography that utilizes finite deformations imaged by dualMRI does not require wave propagation and specialized gradient coils, and therefore provides a means to study stiff cartilage.

Our objective was to establish and test a workflow, termed *finite deformation elastography*, to quantify stiffness in tissues and biomaterials, with a particular emphasis on articular cartilage and analog hydrogels and biomaterials. We utilize a new combination of dualMRI and topology optimization to provide a general framework capable of broad applicability in numerous materials, and in two- and three-dimensional (2D/3D) configurations of material complexity and heterogeneity. We describe development, validation, and application of steady-state, physiological-magnitude cyclic loading to extract the displacements, followed by inverse modeling to estimate relative stiffness maps.

## Results

**Figure 2:**
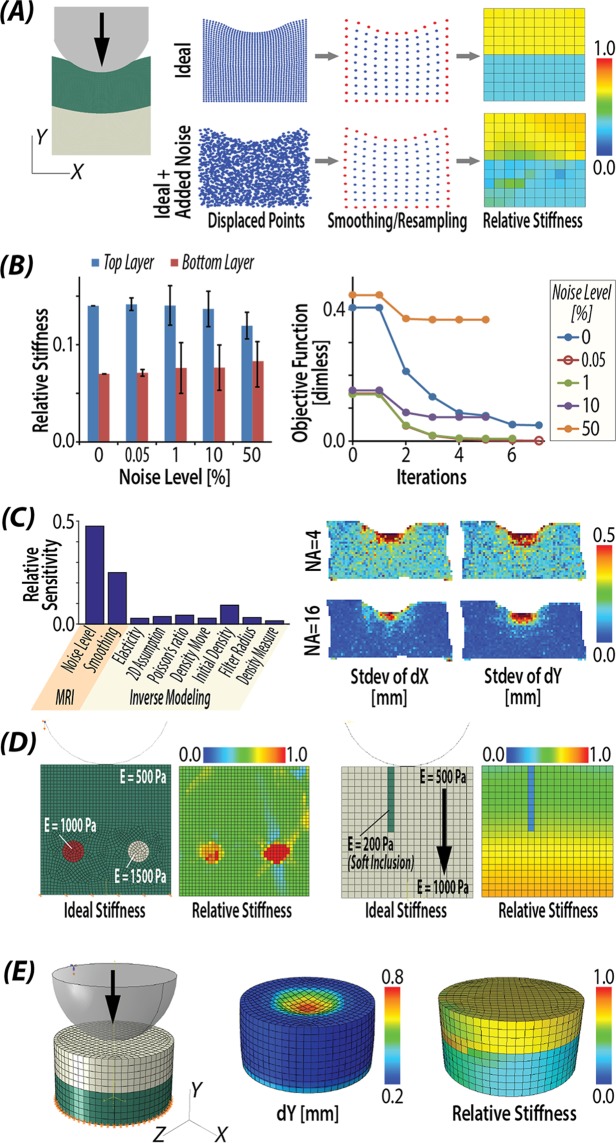
Validation of stiffness reconstructions in complex materials and simulations. (**A**) Stiffness calculation results from ideal displacement with normally distributed noise. (**B**) Stiffness calculation results with standard deviation at different level of noise added; (**C**) Sensitivity values of different factors by Cotter’s method, which identified MRI noise level and smoothing, or the quality of fundamental image data as factors most impacting stiffness measurements. (**D**) Stiffness reconstruction results were robust to inclusion of complex stiff/soft inclusion representative of tissue defects and heterogeneity. (**E**) Bilayer stiffness was reconstructed from displacement in a 3D cylindrical indentation model.

**Figure 3:**
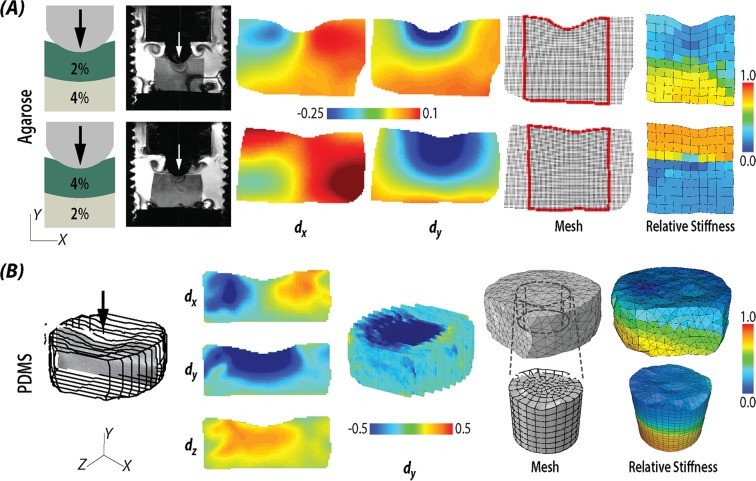
Stiffness reconstruction in multilayered biomaterials. (**A**) Stiffness reconstruction results from bilayer agarose hydrogels with different configurations, including soft over stiff (2% over 4%) gels, and stiff over soft (4% over 2%) gels. (**B**) Stiffness reconstruction results from bilayer PDMS gel in three dimensions.

Finite deformation elastography, based on dualMRI and topology optimization, allowed for inverse calculation of stiffness in cartilage tissues and biomaterials (Fig. 1). Image acquisition was performed in 2D or 3D to provide spatially complex input data. When combined with boundary conditions, topology optimization allowed for reconstruction of stiffness maps under ideal (noise-free) conditions (Fig. 2), and with increasing noise levels and material complexity (Fig. 3).

### Error analysis and sensitivity

The inverse modeling successfully reconstructed the stiffness distribution defined in the forward simulation (Fig. 2A). With the normally distributed error of standard deviation 0.1 *mm* added to the ideal displacement, similar to noise observed in MRI data, the algorithm was still able to reconstruct a bilayer pattern. Moreover, the stiffness calculation was robust to increasing noise levels from the MRI acquisition (Fig. 2B). The defined (2:1) stiffness ratio between two layers decreased as the noise level approached 50%, and the stiffness values in each layer exhibited increased scattering. The objective function corresponding to each noise level decreased with iterations, indicating that the difference of displacements between experiments and simulation was minimized.

The results of the sensitivity analysis, using Cotter’s method, demonstrated that the noise and smoothing technique were the most important (concerning) parameters (Fig. 2C). This emphasized the necessity to improve the SNR of dualMRI and utilize best smoothing techniques to filter the displacement input. One manner to improve the SNR is by increasing the number of averages over which an experiment is repeated. As presented in (Fig. 2C) right panel, increasing the number of averages from 4 to 16 reduced the standard deviation of dX and dY from 0.2 *mm* to 0.1 *mm*. For the smoothing technique, as opposed to Gaussian smoothing, which altered the gradient at the edges for thin masks, LOWESS smoothing did not exhibit a systematic bias, allowing for visualization of through-thickness displacement patterns which reflect cartilage-cartilage contact^28^ (Supplemental Fig. 1). Interestingly, the use of different constitutive laws, including linear elastic, or Neo-Hookean or Mooney-Rivlin solids, indicated minimal influence on the stiffness values (Supplemental Fig. 2).

Stiffness distributions were reliably reconstructed in materials with complex stiffness abnormalities, including (stiff) circular inclusions, gradients of elastic properties, or soft, slender inclusion (Fig. 2D). Additionally, though the structural mesh in the base model was different from the input displacement map, we observed that the method was able to interpolate the meshes and reconstruct the stiffness distribution. Our approach was also validated in three-dimensional loading configurations with a defined 2:1 (top:bottom) stiffness ratio (Fig. 2E). Finally, Monte Carlo simulations revealed a relative stiffness bias of 0.092 and precision of 0.066. An average bias map showed elevated values at the interface of this bi-layer structure and at the bottom where boundary was fixed (Supplemental Fig. 3).

### Stiffness reconstruction of bilayered gel materials

Stiffness distributions were reconstructed in bilayer hydrogels and silicone materials, in both 2D and 3D (Fig. 3). We consistently documented increased stiffness in regions of higher agarose concentrations. We calculated the stiffness ratio between the top and bottom layers and rescaled them as 1:2.0 for the 2%:4% (top:bottom) layered hydrogel, and 3.1:1 for the 4%:2% layered hydrogel. We additionally demonstrated reconstruction of cylindrical (volumetric) stiffness based on 3D MRI displacement data. However, we observed stiffness artifacts (aberrant values) arising from dualMRI near the edges (Supplemental Fig. 4), and proposed two solutions to overcome potential problems (Fig. 3B). In one solution, the entire data space is used with the addition of a larger filter radius. In a second solution, the artifact regions were removed to create a partial model with boundary conditions defined at new edges. As it has been demonstrated with ideal forward simulation displacements (Fig. 2E), the bilayer cylinder was successful restored with both methods.

### Elastography in articular cartilage within intact tibiofemoral joints

**Figure 4:**
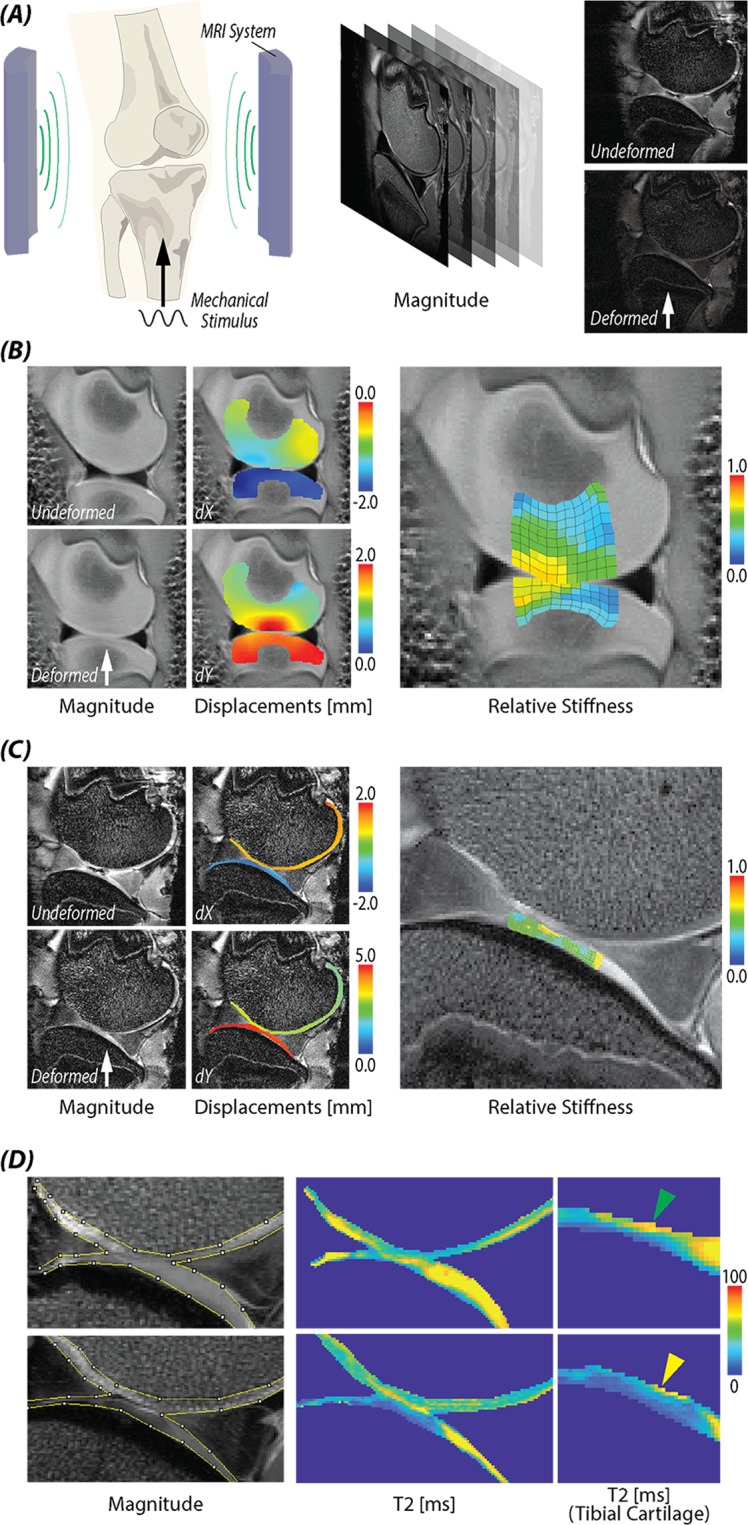
Stiffening of the articular cartilage surface zone within intact tibiofemoral joints under cyclic loading. (**A**) Experimental setup of knee joint loading within an MRI system. (**B**) A juvenile porcine knee joint was loaded to noninvasively measure displacements and calculate relative stiffnesses. (**C)** An adult sheep knee was loaded to measure displacement and stiffness of cartilage, revealing increased stiffness at the articular surface. (**D**) *T*_2_ value in cartilage before and after loading supported the increased stiffness measurement, and indicated water depletion and cartilage densification that likely occurred during cyclic loading before (preconditioning) and during image acquisition.

We were able to further calculate stiffness values within the articular cartilage of intact tibiofemoral joints (Fig. 4). In an intact porcine joint (Fig. 4B), while the maximum displacements in the loading direction (dY) were greatest in the superficial region of femoral cartilage, the relative stiffness in the superficial zone was observed to be stiffer than middle or deep cartilage zones (nearer to subchondral bone). In sheep joint cartilage, a stiffer superficial layer was also observed in the tibia cartilage, compared to middle or deep zones (Fig. 4C). We observed a decrease in *T*_2_ values in the superficial region that corresponded to regions of elevated stiffness (Fig. 4D). We additionally note that the values for transverse relaxation time *T*_2_ are very sensitive to water content in cartilage^29^.

## Discussion

The purpose of this study was to develop *finite deformation elastography*, a hybrid of magnetic resonance imaging and topology optimization, to investigate stiffness heterogeneity, including spatial gradients and inclusions, in soft tissues and biomaterials. To solve for stiffness distribution from displacement fields, inverse methods based on a complete understanding of the equivalent forward simulation was required. The forward elasticity problem was usually described by numerical techniques such as the finite difference method^30–32^, and the finite element method^33–35^, with the latter preferred due to its ability to tackle complex geometries, inhomogeneities, and boundary conditions. To reconstruct stiffness distributions from the forward models, indirect iterative methods are preferably used, compared to direct inversion of matrices, by updating the stiffness distribution to minimize the fit errors between the simulated model and the experimental displacement/strain results^35,36^. In this study, we utilized an iterative finite element method to reconstruct stiffness matrices using topology optimization^36^. With this framework, displacement or stress boundary conditions can be specified^37^, and the stiffness distribution is iteratively updated to match the measured displacements as design objectives.

Validation studies revealed that our approach was able to reconstruct heterogeneous stiffness distributions, including a bilayer configuration of hydrogels and silicone materials, and in materials with stiff circular and soft slender inclusions. These patterns specifically mimicked different stiffness patterns that could exist in healthy and diseased tissues like articular cartilage (e.g. vertical fissures as soft inclusions when the depth or strain response under mechanical loading can be captured by MRI^13^), and are more broadly representative of a wide range of tissues and materials exhibiting gradient and nonuniform stiffness distributions. Moreover, our validation studies revealed that inverse modeling was robust to increasing noise levels. Taken together, the hybrid combination of imaging and topology optimization represents an effective means to reconstruct unknown interior stiffness distributions of complex materials based on noninvasive imaging.

Sensitivity analysis (via Cotter’s method) indicated that the noise level and the smoothing technique were the most significant factors impacting the technique error. To improve the SNR of the dualMRI system, the number of averages can be used at the cost of longer total imaging time. SNR could additionally be improved by minimizing the loading time to rapidly capture the encoded prior to signal loss, which follows an exponential *T*_1_ (~sec) decay. To improve the smoothing technique, we filtered the noise in displacement data using LOWESS, which demonstrated better localized fitting of noise compared to Gaussian fitting^27^, especially at material edges. Monte Carlo simulation of smoothing techniques showed a smaller bias and precision using LOWESS smoothing. Moreover, the calculated bias of 0.092 and precision of 0.066 were approximately 18% and 13.2%, respectively of the average relative stiffness (of 0.5; Fig. 2B). Considering the noise level of 0.1 mm (20% with average 0.5 mm deformation), the stiffness reconstruction method did not deteriorate the data quality. Based on this analysis, it is advantageous to set image acquisition parameters that maximize image quality (e.g. with maximum SNR) to ensure that minimal error propagates through topology optimization to influence stiffness measures.

Finite deformation elastography successfully reconstructed the bilayer pattern of stiffness in hydrogels and biomaterials. In 2D studies, the stiffness ratio between top and bottom layer were calculated to be 1:2.0 for the first case with 2% layer on top and 1:3.1 for the latter case with 4% on top. As a comparison, an unconfined compression test measured the instantaneous modulus at largest stress of 2% and 4% agarose gels to be 74.6 kPa and 173.4 kPa respectively (1:2.3 ratio), and equilibrium modulus after 30 *sec* relaxation to be 23 kPa and 112.7 kPa respectively (1:4.9 ratio)^27^. In both stiffness bi-layer configurations, the inverse-calculated stiffness ratio was close to the bulk testing results. Specifically, the 1:2.0 ratio derived from soft top – stiff bottom configuration was closer to the instantaneous modulus ratio of 1:2.3 than the stiff top – soft bottom configuration. This could be due to the error of the plane stress assumption, since this was a 3D cylindrical gel fixed at the bottom but unconfined at the side. In that way, the out of plane constraints behaves differently in bottom and top and might cause the ratio of stiffness to be different in these two reversed configurations. Besides, the cylindrical indenter should align well with the gel center in ideal scenario, but any error could generate a bias for through-thickness differences. The 3D indentation case showed that with the acquisition of multiple slices of displacement data, it was possible to reconstruct the stiffness distribution without the 2D assumption. Consequently, our workflow was not constrained by complex geometries. Potentially, since topology optimization can also be utilized in contact problems, it is possible to calculate relative stiffness in multiple objects in contact, like the femur and tibia cartilage.

Topology optimization is similar to several iterative techniques that reconstruct the stiffness distribution^35,38^ utilizing (1) a forward elasticity problem and finite element analysis, and (2) iterative updates to the stiffness distribution by minimizing the measured displacement distribution. Compared to these techniques, our topology optimization-based method is more computationally efficient and can accommodate different constitutive models including linear elasticity and hyperelasticity, as well as material anisotropies (Supplemental Fig. 2). It is also possible to model complex geometries and boundary conditions, and enable conservative or aggressive update strategies^36^. For concerns of outliers in the displacement input data, local regression smoothing is used prior to inverse modeling. Additionally, predetermined weight factors can be added to the design objective based on the input displacement smoothness. Currently, the design objective sensitivity is filtered to help to regularize the problem and to make the algorithm mesh independent and converge faster^39^. If direct filtering on stiffness value is desired, the weight factor on each node can be adjusted at each iteration, based on the smoothness of stiffness map.

Because our current framework utilized displacement boundary conditions, we focused on the calculation of relative stiffness. Compared to stress boundary conditions, displacement boundary conditions are independent of an initial guess, and produce superior images in terms of both spatial resolution and stiffness measurement sensitivity^37^. Importantly, depending on the experimental measurements available, our inverse simulation method can be used to solve stress or displacement boundary conditions, or a hybrid of both. In future embodiments, inclusion of stress boundary conditions can enable calculation of absolute stiffness through the material interior. Additionally, it is important to note that finite element analysis software was used here largely because of its strong nonlinear capability and wide applications in biomechanics field^36^. However, topology optimization is not restricted to any specific software.

In our porcine and sheep joint loading experiments, stiffness distributions were reconstructed for cartilage within the articular joint space *in situ*. Due to the long scanning time (~30 *min* for each slice for these data), only a single slice was acquired using dualMRI. Additionally, repeated loading of the cartilage surface was advantageous in our case as it led to collagen fiber compaction and reorientation representing the material response to physiologically-relevant loading, and a stiffer superficial layer^40^. Cyclic loading before imaging (i.e., preconditioning), and during imaging, likely led to lateral tissue deformation and fluid flow, particularly in the superficial zone, further exasperating collagen densification after cyclic loading^41^. The loading and flow resulted in compaction as a temporary change, similar to what may be expected during a walking cycle, which would be restored by Donnan equilibrium after cyclic loading is ended. Our observations of a stiffer superficial layer, and assumptions of fiber compaction and water loss, were supported by decreased *T*_2_ magnitudes in the superficial layer after loading^42–44^. Additionally, while cyclic loading was acquired over a long total experimental time, the loading rate within any cycle was considered rapid (0.3 sec to reach one-times body weight), and a nearly incompressible behavior was assumed. Importantly, the compressibility assumption in our workflow relates to potential errors in estimates for mechanical parameters (Supplemental Fig. 2), which indicates that for a broad range of (e.g. quasi-static) loading conditions, the assumption of incompressibility requires further study.

Our *finite deformation elastography* workflow, which was based on dualMRI and topology optimization, enabled the measurement of stiffness within musculoskeletal tissues and biomaterials. Compared to traditional MRE, which excites and images shear wave maps to calculate shear stiffness, our elasticity reconstruction technique does not depend on shear waves, and thus is capable of measurements in stiff structures or tissues with complex interfaces, including bony articulating joints of the body. Sensitivity analysis indicated that MRI acquisition, and not topology optimization, parameters dominate the error in the workflow, and suggest that improvements in MRI SNR should remain as a primary goal when setting experimental parameters to have the greatest potential to improve stiffness measures. Moreover, Monte Carlo simulations were utilized to evaluate the bias and precision of the stiffness reconstruction, which was measured to be no larger than the input displacement measurements. We also extended this technique to three dimensions, showing that our approach was not constrained by plane stress/strain assumptions or complex geometries. We additionally tested the elastography approach on sheep and porcine joint articular cartilage *in situ*, reconstructed stiffness maps in cartilage at the submillimeter scale, and found that the superficial layer only was densified after cyclic loading and during image acquisition. We envision that our technique can be potentially be used to analyze cartilage softening observed in osteoarthritis or damage, similar to data presented on stiffness inclusions, or provide a unique imaging biomarker for tissue repair. In order to reach the goal of *in vivo*, first-in-human elastography measurements, improvements to reduce the imaging acquisition time (e.g. through rapid MR acquisition) and additional *in situ* studies on the effects of preconditioning of healthy and degraded tissue are required. Additionally, finite deformation elastography can be extended to other load-bearing and stiff biomaterials of the musculoskeletal system, including intervertebral disc and ligament.

## Methods

Finite deformation elastography is based on dualMRI to measure displacements during cyclic loading and topology optimization to calculate internal patterns of stiffness. To develop and validate our workflow, we evaluated how displacement maps determined by experiment or simulation, with predefined stiffness distributions, were used with topology optimization to reconstruct stiffness values. We further evaluated how the sensitivity of a broad space of independent variables (e.g. levels of random noise) influenced method robustness, and to understand how error propagates through the calculation workflow. Using finite deformation elastography, we measured stiffness in unique hydrogel and material model systems in two and three dimensions, and for the first time in cartilage *in situ*, deep within the bony contacting interface of porcine and sheep joints.

### Finite deformation elastography workflow

#### Imaging high-magnitude displacements at low frequency

Using dualMRI, (Fig. 1B), samples were cyclically loaded at high (e.g. ~0.1–10 mm displacement; up to 1–2 times body weight) magnitude and low (e.g. ~0.1–1.0 Hz) frequency. To acquire displacements and visualize internal sample motion, we used an MRI pulse sequence that incorporated displacement encoding with a stimulated echo (DENSE) imaging^39^. The DENSE sequence sensitized the phase data Δφ to changes in displacement, Δx, according to:

1 $$\Delta {\boldsymbol{\varphi }}={{\boldsymbol{\gamma }}}_{{\boldsymbol{H}}}{{\boldsymbol{t}}}_{{\bf{e}}{\bf{n}}{\bf{c}}}({{\boldsymbol{G}}}_{{\bf{d}}{\bf{e}}}-{\boldsymbol{G}}{\text{'}}_{{\bf{d}}{\bf{e}}})\Delta {\boldsymbol{x}}{\boldsymbol{,}}$$

where ${\gamma }_{H}$ is the gyromagnetic ratio, ${t}_{{\rm{enc}}}$ is the duration of the encoding gradient, ${G}_{{\rm{de}}}$ is the gradient magnitude for *xyz* displacement encoding, and ${G{\prime} }_{{\rm{de}}}$ is the gradient magnitude for a reference image used to eliminate other phase contributions common to both images^25,45^. The whole displacement measurement included phase mapping, phase unwrapping to calculate raw displacement, and displacement smoothing as described previously (Fig. 1C, Supplemental Fig. 1)^27^.

#### Reconstruction of stiffness distributions

To inversely calculate the stiffness map from displacements, we utilized topology optimization (Fig. 1D). Traditionally, topology optimization is a non-parametric optimization method to design stiff, durable and light-weight structures^25^. Here, displacement boundary conditions were used, which can be readily adapted to alternatively include stress boundary conditions^37^. Compared to the previous iterative FE methods which use Newton-Raphson or Gauss-Newton methods^39^, topology optimization uses the method of moving asymptotes, which convert each single iteration into a subproblem with separable and convex approximations^46–48^. This method generally reduced the computational effort and made it possible to deal with models that included a large number of design variables, defined here as element stiffness, and additionally with constraints such as stiffness limits and smoothness^35^. Topology optimization was also not constrained by the constitutive laws of different materials, and can be applied with linear elastic, hyperelastic, and anisotropic materials^37^, and also in problems where with nonlinearities in geometry, material behavior, or contact.

In our elastography workflow, instead of maximizing the structure stiffness, as is used in traditional applications, we minimized the maximum value of the absolute difference between the measured material deformation and the deformation of a base model at nodes indicated as *P*_*i*_^37^:

2 $$F=\,{\rm{\min }}:{{\rm{\max }}}_{i=1}^{N}(|\Delta {x}_{\exp }({P}_{i})-\Delta {x}_{{\rm{sim}}}({P}_{i})|)$$

where *F* is the design objective and Δ*x*_*exp*_, Δ*x*_*sim*_ are experimental, simulated displacement at internal node *P*_*i*_. If outliers exist in the displacements measured, smaller weights are applied beforehand on those nodes. The finite deformation elastography workflow was solved using finite element method software (Abaqus, Version 2017)^37^ and optimization software (Tosca, Version 2017; Dassault Systèmes)^39^, to iteratively update the stiffness of each element representing the material as described (Supplemental Materials). Briefly, the stiffness of each element was associated with a relative density parameter ρ_*k*_, and the material properties of a given material ${E}^{0}$, through the Solid Isotropic Material with Penalization (SIMP) method^49^:

3 $$\begin{array}{l}{E}_{k}={({\rho }_{k})}^{p}{E}^{0}\\ 0 < {\rho }_{{\rm{\min }}} < {\rho }_{k} < 1\end{array}\}$$

Instead of penalizing the density results to reach a 0 (void) to 1 (solid) range, we set the $p=1$ to make the stiffness of the constitutive material modeling linearly related with the density in element *k*. In this study, if not specified, a linear elastic model with Poisson’s ratio 0.49 was used. Because we utilized a displacement boundary simulation, a random Young’s modulus 500 was chosen. For element types, CPS8 was used for 2D meshes, and tetrahedral element C3D4 and brick element C3D8 were used for 3D meshes. A mesh density of 0.6 mm was usually chosen to demonstrate the stiffness heterogeneity and at the same time to keep the computational cost low.

Following determination of displacements, 2D and 3D models were established using the location of each pixel in the model or sample (MATLAB), and Delaunay triangulations were created to connect each pixel. The boundaries of the model were automatically identified with positive area or volume triangulation, and with a dimension not shorter than 0.5 *mm*. In our finite element models, displacements in all coordinates were predefined at all pixels at boundaries.

### Error analysis and sensitivity

Error analysis and sensitivity studies were performed to validate finite deformation elastography. We first validated our workflow in two dimensions, with ideal displacements that were derived from forward modeling in finite element software. A rectangular model of side length 6 by 6 *mm* was created (Fig. 2A) with a bilayer stiffness structure defined by a top and bottom stiffness of E = 1000 *Pa* and 500 *Pa*, respectively. The element size matched the dualMRI spatial resolution (i.e., 150 *μm*). Considering the rapid (0.3 *sec*) loading ramp and the large water content of the real cartilage, fluid permeation is very minimal by Darcy’s law^25^ and nearly incompressible behavior (Poisson’s ratio = 0.49) was assumed^27^. With the bottom edge of the model fully constrained, the top edge was indented to 15% of the thickness, and displacements were calculated, with linear elasticity and plane stress assumptions. To analyze the robustness of the algorithm against noise, different levels of noise were applied and the average stiffness of two layers were calculated (Fig. 2B).

We ranked the model input parameters based on their influence on the model output using a sensitivity analysis (Cotter’s method)^49^. A two-level, factorial design was used with all parameters set either at an extreme high or low level. We considered the following parameters: noise level in the raw input data, smoothing technique applied, and FE modeling parameters such as linear/hyperelastic (i.e., linear elastic, Neo-Hookean, or Mooney-Rivlin) constitutive laws, Poisson’s ratio, 2D assumption, and optimization values. In this analysis, Gaussian smoothing and LOcally WEighted Scatterplot Smoothing (LOWESS) were compared and the analysis provided a ranking for each factor by its impact to the final output.

To analyze the stiffness error (defined by precision and bias) of the whole elastography procedure, we applied Monte Carlo simulations. The pixel number, model dimensions, and data noise (standard deviation = 0.1 *mm*), were determined from displacement-encoded MRI data^27^. Within each iteration of the Monte Carlo simulation, experimental level Gaussian random noise (standard deviation = 0.1 *mm*) was added directly to the ideal displacement not only the internal pixels but also pixels at boundaries. By repeating the random noise generation and inverse simulation for 100 times^50^, different stiffness maps were reconstructed. Precision was defined as the pooled standard deviation of respective stiffness from all elements between the reconstructed map and the distribution from the ideal model. Bias was calculated by determining the root mean square error of the stiffness value.

We further validated our ability to reconstruct stiffness maps in model systems representative of variation expected in biological samples. Using a 2D model, similar to (Fig. 2A) with a homogeneous stiffness (500 Pa), we added stiff (1000 and 1500 Pa) inclusions representative of tissue heterogeneity during disease or degeneration (Fig. 2D). In a separate model, we additionally added a soft (200 Pa) inclusion, similar to a tissue fissure, on a background of a gradient increasing stiffness (to 1000 Pa) (Fig. 2D). Finally, we simulated a cylindrical model to reconstruct a 3D stiffness map (Fig. 2E).

### Stiffness reconstruction of bilayered gel materials

To validate finite deformation elastography in complex engineered materials, we prepared multiple bilayer models with defined stiffness values in top and bottom layers. In a hydrogel model system^51^, 2% (softer) and 4% (stiffer) w/v agarose was mixed in PBS and cured to create uniform and layered constructs with a 6 *mm* total height, and 10 *mm* diameter cylindrical implants. There were two gels made to test the stiffness reconstruction accuracy: one gel had 2% agarose (soft) on the top with 2/3 thickness and the second gel was created with 4% agarose (stiff) on the top with 1/3 thickness. In a separate silicone model system, polydimethylsiloxane (PDMS) (Dow Corning, Midland, MI)^38^ was used to create a bilayer gel with a 1:2 mix ratio (Sylgard 527) in the top (soft) layer, and a 1:50 mix ratio (Sylgard 184) in the bottom (stiff) layer. These materials allowed us to establish baseline deformation and noise levels commonly observed in small materials or explanted tissues^52^ (Fig. 3A).

Finite displacements were determined using dualMRI^10^ under cyclic compressive loading with a DENSE-FISP imaging sequence at 0.33 *Hz* frequency, and with a spatial resolution of 0.10 × 0.10 *mm*^2^ (agarose) or 0.15 × 0.15 *mm*^2^ (silicone). For hydrogel samples, the target load was set to be 0.67 *N* and the gel reached indentation of 6% (configuration: 2% agarose on the top) and 4.6% (configuration: 4% agarose on the top) of the sample depth. As a two-dimensional case, plane stress was assumed for agarose gel and displacement data from the middle section was extracted. For silicone samples, to address the potential bias of the 2D assumption mentioned in the sensitivity analysis, inverse modeling was tested in three dimensions. To test the algorithm on experimental data, we applied 0.33 *Hz* frequency load to reach 15% indentation and collected the *xyz* displacement data from 13 consecutive slices (Supplemental Fig. 4). As a 3D linear elastic model, tetrahedral element C3D4 and brick element C3D8 were used. Since the hydrogel and PDMS materials were considered to be incompressible^53,54^, a Poisson’s ratio 0.49 was used. Prior to topology optimization, displacements were smoothed using LOWESS in MATLAB (Supplemental Fig. 1)^27^. For smoothing by LOWESS, least absolute residuals were utilized to make the process resistant to outliers due to the noise. The window size for calculating local weight was set to be 150 pixels for consistency among different regions of interest. Finally, we compared different material models, using displacement data from a homogeneous PDMS gel (Supplemental Fig. 2) and linear elastic, Neo-Hookean, or Mooney-Rivlin constitutive models.

### Elastography in articular cartilage within intact tibiofemoral joints

To demonstrate finite deformation elastography in a challenging biological system, we measured stiffness in the articular cartilage of intact tibiofemoral joints. dualMRI data describing internal displacements of cartilage juvenile porcine joints^38^ and sheep joints^55^ were utilized from previous studies. Both data sets were acquired after preconditioning and during cyclic loading at a spatial resolution of 0.25 × 0.25 *mm*^2^, with displacements resulting from 0.2 *Hz* frequency loading at one-times body weight (78 *N* for a 2-month old porcine and 445 *N* for an adult sheep). We note that the loading frequency was tuned for ramp loading (i.e., ramp to load, ramp to unload), which resulted in a steady-state response^56^. As explained previously, the material was modeled as nearly incompressible material with Poisson’s ratio 0.49, considering the time duration needed to reach (i.e., ramp to) one-times body weight in each loading cycle was only 0.3 sec. Additionally, because we observed a counterintuitive stiffening of the cartilage in the superficial zone (described subsequently), we also acquired an independent measurement of matrix structure using MRI *T*_2_ relaxometry mapping. *T*_2_ was determined at each volumetric region using monoexponential fitting of data from a multi-echo sequence with parameters: TE/TR = 10.04/4000 *ms*, number of averages = 1, rare factor = 2, echo spacing = 20.08 *ms*.

### Statistics

One-way Analysis of Variance (ANOVA), followed by post hoc Tukey’s test was used to determine statistically significant differences between the groups. The coefficient of regression (*R*^2^) was calculated using linear regression.

## Supplementary information

Supplementary Information.

## Data Availability

The datasets generated during and/or analyzed during the current study are available from the corresponding author on reasonable request.

## References

[1] Wells RG (2005). The role of matrix stiffness in hepatic stellate cell activation and liver fibrosis. Journal of clinical gastroenterology.

[2] Wellman, P., Howe, R. D., Dalton, E. & Kern, K. A. Breast tissue stiffness in compression is correlated to histological diagnosis. Harvard BioRobotics Laboratory Technical Report, 1–15 (1999).

[3] Liu T, Babaniyi OA, Hall TJ, Barbone PE, Oberai AA (2015). Noninvasive *in-vivo* quantification of mechanical heterogeneity of invasive breast carcinomas. PLoS One.

[4] Friedl P, Alexander S (2011). Cancer invasion and the microenvironment: plasticity and reciprocity. Cell.

[5] Mierke CT (2012). Endothelial cell’s biomechanical properties are regulated by invasive cancer cells. Molecular BioSystems.

[6] Yelin E (2007). Medical care expenditures and earnings losses among persons with arthritis and other rheumatic conditions in 2003, and comparisons with 1997. Arthritis & Rheumatology.

[7] Neu C (2014). Functional imaging in OA: role of imaging in the evaluation of tissue biomechanics. Osteoarthr. Cartilage.

[8] Gomoll A (2012). Surgical treatment for early osteoarthritis. Part I: cartilage repair procedures. Knee Surg. Sport Tr. A..

[9] Kon E (2012). Non-surgical management of early knee osteoarthritis. Knee Surg. Sport Tr. A..

[10] Griebel AJ, Trippel SB, Emery NC, Neu CP (2014). Noninvasive assessment of osteoarthritis severity in human explants by multicontrast MRI. Magn. Reson. Med..

[11] Kempson G, Muir H, Pollard C, Tuke M (1973). The tensile properties of the cartilage of human femoral condyles related to the content of collagen and glycosaminoglycans. Biochimica et Biophysica Acta (BBA)-General Subjects.

[12] Hayes W, Mockros L (1971). Viscoelastic properties of human articular cartilage. J. Appl. Physiol..

[13] Waldstein W (2016). OARSI osteoarthritis cartilage histopathology assessment system: a biomechanical evaluation in the human knee. J. Orth. Res..

[14] Setton LA, Elliott DM, Mow VC (1999). Altered mechanics of cartilage with osteoarthritis: human osteoarthritis and an experimental model of joint degeneration. Osteoarthr. Cartilage.

[15] Kim W, Ferguson VL, Borden M, Neu CP (2016). Application of elastography for the noninvasive assessment of biomechanics in engineered biomaterials and tissues. Ann. Biomed. Eng..

[16] Zheng Y-P (2004). High resolution ultrasound elastomicroscopy imaging of soft tissues: system development and feasibility. Phys. Med. Biol..

[17] Laasanen MS (2003). Ultrasound indentation of bovine knee articular cartilage *in situ*. J. Biomech..

[18] Nieminen HJ (2002). Real-time ultrasound analysis of articular cartilage degradation *in vitro*. Ultrasound Med. Biol..

[19] Muthupillai R (1995). Magnetic resonance elastography by direct visualization of propagating acoustic strain waves. Science.

[20] Lopez O, Amrami KK, Manduca A, Rossman PJ, Ehman RL (2007). Developments in dynamic MR elastography for in vitro biomechanical assessment of hyaline cartilage under high‐frequency cyclical shear. J. Magn. Reson. Imaging.

[21] Faber S (2001). Gender differences in knee joint cartilage thickness, volume and articular surface areas: assessment with quantitative three-dimensional MR imaging. Skeletal Radiol..

[22] Lopez O, Amrami KK, Manduca A, Ehman RL (2008). Characterization of the dynamic shear properties of hyaline cartilage using high-frequency dynamic MR elastography. Magn. Reson. Med..

[23] Streitberger KJ (2015). *In vivo* multifrequency magnetic resonance elastography of the human intervertebral disk. Magn. Reson. Med..

[24] Chan DD, Neu CP (2012). Transient and microscale deformations and strains measured under exogenous loading by noninvasive magnetic resonance. PloS one.

[25] Neu CP, Walton JH (2008). Displacement encoding for the measurement of cartilage deformation. Magn. Reson. Med..

[26] Chan DD (2016). *In vivo* articular cartilage deformation: noninvasive quantification of intratissue strain during joint contact in the human knee. Sci. Rep..

[27] Chan DD (2018). Functional MRI can detect changes in intratissue strains in a full thickness and critical sized ovine cartilage defect model. J. Biomech..

[28] Butz, K. D. Numerical techniques for the noninvasive assessment of material properties and stresses in soft biomaterials. (2013).

[29] Lüssea S (2000). Evaluation of water content by spatially resolved transverse relaxation times of human articular cartilage. Magn. Reson. Imaging.

[30] Chan DD, Neu CP (2013). Intervertebral disc internal deformation measured by displacements under applied loading with MRI at 3T. Magn. Reson. Med..

[31] Skovoroda A, Emelianov S, o’Donnell M (1995). Tissue elasticity reconstruction based on ultrasonic displacement and strain images. Ultrasonics, Ferroelectrics and Frequency Control, IEEE Transactions on.

[32] Raghavan K, Yagle AE (1994). Forward and inverse problems in elasticity imaging of soft tissues. Nuclear Science, IEEE Transactions on.

[33] Zhu Y, Hall TJ, Jiang J (2003). A finite-element approach for Young’s modulus reconstruction. Medical Imaging, IEEE Transactions on.

[34] Romano AJ, Shirron JJ, Bucaro JA (1998). On the noninvasive determination of material parameters from a knowledge of elastic displacements theory and numerical simulation. Ultrasonics, Ferroelectrics and Frequency Control, IEEE Transactions on.

[35] Kallel F, Bertrand M (1996). Tissue elasticity reconstruction using linear perturbation method. Medical Imaging, IEEE Transactions on.

[36] Doyley M, Meaney P, Bamber J (2000). Evaluation of an iterative reconstruction method for quantitative elastography. Phys. Med. Biol..

[37] Bendsoe, M. P. & Sigmund, O. Topology optimization: theory, methods and applications. (Springer, 2003).

[38] Griebel A, Khoshgoftar M, Novak T, van Donkelaar C, Neu C (2014). Direct noninvasive measurement and numerical modeling of depth-dependent strains in layered agarose constructs. J. Biomech..

[39] Systèmes, D. In *Simulia Corp. Providence, RI, USA* (2017).

[40] Ateshian GA, Maas S, Weiss JA (2013). Multiphasic finite element framework for modeling hydrated mixtures with multiple neutral and charged solutes. J. Biomech. Eng..

[41] Kaplan JT, Neu CP, Drissi H, Emery NC, Pierce DM (2017). Cyclic loading of human articular cartilage: the transition from compaction to fatigue. J. Mech. Behav. Biomed. Mater..

[42] Alhadlaq HA, Xia Y (2005). Modifications of orientational dependence of microscopic magnetic resonance imaging T2 anisotropy in compressed articular cartilage. Journal of Magnetic Resonance Imaging: An Official Journal of the International Society for Magnetic Resonance in Medicine.

[43] Gründer W, Kanowski M, Wagner M, Werner A (2000). Visualization of pressure distribution within loaded joint cartilage by application of angle‐sensitive NMR microscopy. Magnetic resonance in medicine.

[44] Nag D, Liney GP, Gillespie P, Sherman KP (2004). Quantification of T2 relaxation changes in articular cartilage with *in situ* mechanical loading of the knee. Journal of Magnetic Resonance Imaging: An Official Journal of the International Society for Magnetic Resonance in Medicine.

[45] Aletras AH, Ding S, Balaban RS, Wen H (1999). DENSE: displacement encoding with stimulated echoes in cardiac functional MRI. J. Magn. Reson..

[46] Bendsøe MP, Sigmund O (1999). Material interpolation schemes in topology optimization. Archive of applied mechanics.

[47] Svanberg K (1987). The method of moving asymptotes—a new method for structural optimization. International journal for numerical methods in engineering.

[48] Svanberg K (2002). A class of globally convergent optimization methods based on conservative convex separable approximations. SIAM journal on optimization.

[49] Systèmes, D. TOSCA Structure manual. www.simulia.com (2016).

[50] Neu C, Hull M, Walton J (2005). Heterogeneous three‐dimensional strain fields during unconfined cyclic compression in bovine articular cartilage explants. J. Orth. Res..

[51] Neu CP, Hull ML, Walton JH (2005). Error optimization of a three-dimensional magnetic resonance imaging tagging-based cartilage deformation technique. Magn. Reson. Med..

[52] Chan DD, Toribio D, Neu CP (2013). Displacement smoothing for the precise MRI-based measurement of strain in soft biological tissues. Comput. Methods Biomech. Biomed. Eng..

[53] Anseth KS, Bowman CN, Brannon-Peppas L (1996). Mechanical properties of hydrogels and their experimental determination. Biomaterials.

[54] Pritchard RH, Lava P, Debruyne D, Terentjev EM (2013). Precise determination of the Poisson ratio in soft materials with 2D digital image correlation. Soft Matter.

[55] Chan DD, Neu CP, Hull ML (2009). Articular cartilage deformation determined in an intact tibiofemoral joint by displacement‐encoded imaging. Magn. Reson. Med..

[56] Chan D, Neu C, Hull M (2009). *In situ* deformation of cartilage in cyclically loaded tibiofemoral joints by displacement-encoded MRI. Osteoarthr. Cartilage.

[57] Mariappan YK, Glaser KJ, Ehman RL (2010). Magnetic resonance elastography: a review. Clin. Anat..

