# Supplementary material for: Finite deformation elastography of articular cartilage and biomaterials based on imaging and topology optimization
Source: Sci Rep. 2020 May 14;10:7980. doi: 10.1038/s41598-020-64723-9 (PMC7224212; doi:10.1038/s41598-020-64723-9)
Supplement: Supplementary file 1 — Supplementary Information. [file 41598_2020_64723_MOESM1_ESM.pdf]

SUPPLEMENTAL MATERIALS

Finite deformation elastography of articular cartilage and biomaterials based on imaging and topology optimization

Short title: Imaging and topology optimization for finite deformation elastography

Authors

Luyao Cai[1], Eric A. Nauman[1,2,3], Claus B.W. Pedersen[4], Corey P. Neu[1,5,*]

Affiliations

[1]Weldon School of Biomedical Engineering, Purdue University, West Lafayette, IN, 47907, US
[2]School of Mechanical Engineering, Purdue University, West Lafayette, IN, 47907, US
[3]Department of Basic Medical Sciences, Purdue University, West Lafayette, IN, 47907, US
[4]Dassault Systèmes Deutschland GmbH, 20095, Hamburg, Germany
[5]Department of Mechanical Engineering, University of Colorado Boulder, Boulder, CO, 80309, US

*** Corresponding Author**

Corey P. Neu, 1111 Engineering Drive, UCB 427, Boulder, CO, 80309, USA, Phone: (303) 492-7330, cpneu@colorado.edu

Finite Deformation Elastography and Topology Optimization Workflow

Generally, the finite deformation elastography workflow was solved using finite element method software (Abaqus, version 2017) [38] and optimization software (Tosca, version 2017; Dassault Systèmes) [40], to iteratively update the stiffness of each element representing the material. As illustrated in the main text, the stiffness E_k of each element k was designed to be proportional to the relative element density by SIMP method in equation (3). To solve the optimization problem, we used the method of moving asymptotes [55,56]. The approximation of the design function F of element density $\rho = (\rho_1, \rho_2, \dots, \rho_n)$ around a given iteration point ρ^0 has the form:

$$\boldsymbol{F(\rho) \approx F(\rho^0) + \sum_{i=1}^{N} \left(\frac{r_i}{U_i - \rho_i} + \frac{s_i}{\rho_i - L_i} \right)} \qquad (1)$$

where the number r_i, s_i, are chosen as

$$\text{if } \frac{\partial F}{\partial \rho_i}(\rho^0) > 0 \text{ then } r_i = (U_i - \rho_i^o)^2 \frac{\partial F}{\partial \rho_i}(\rho^0) \text{ and } s_i = 0,$$

$$\text{if } \frac{\partial F}{\partial \rho_i}(\rho^0) < 0 \text{ then } r_i = 0 \text{ and } s_i = -(\rho_i^o - L_i)^2 \frac{\partial F}{\partial \rho_i}(\rho^0),$$

and positive numbers U_i, L_i give vertical asymptotes for the approximation of F and are updated heuristically in each iteration to make the iteration more efficient.

Starting with a homogeneous initial stiffness distribution, the resulting displacements with boundary conditions were calculated. Then the difference of nodal displacements was determined between model and measured results. If only marginal improvement was found in the objective function value compared to the last iteration, such as improvement within 0.1% compared to last iteration by default, the optimization was assumed to be converged and the iterations were stopped. Otherwise, the density in elements was updated and the iterations were continued. To achieve the mesh-independency and computer efficiency, the sensitivity value of the design objective F on the element density ρ_k was filtered [57]:

$$\boldsymbol{\frac{\widehat{\partial F}}{\partial \rho_k} = \frac{1}{\rho_k \sum_{i=1}^{N} \widehat{H}_i} \sum_{i=1}^{N} \widehat{H}_i \, \rho_i \frac{\partial F}{\partial \rho_i}} \qquad (2)$$

The mesh-independent convolution operator $\widehat{H}_i$ which decays linearly with the distance from element k was written to be:

$$\widehat{H}_i = r_{min} - \text{dist}(k, i), \quad (\text{dist}(k, i) \leq r_{min}) \qquad (3)$$

where r_{min} was a user-defined filtering radius. Depending on the element sizes, the filter radius was generally chosen to be twice the mean element edge length. Compared to results obtained by a local gradient constraint, the sensitivity filter provided very similar results and required little extra computational time [38]. To further regularize the problem, measured displacements were also filtered to suppress outliers. Direct filtering on the density/stiffness was also achieved by adding constraints of displacement smoothness for neighboring nodes or customizing weight factors in the objective function to force a smooth distribution.

Supplemental Figures

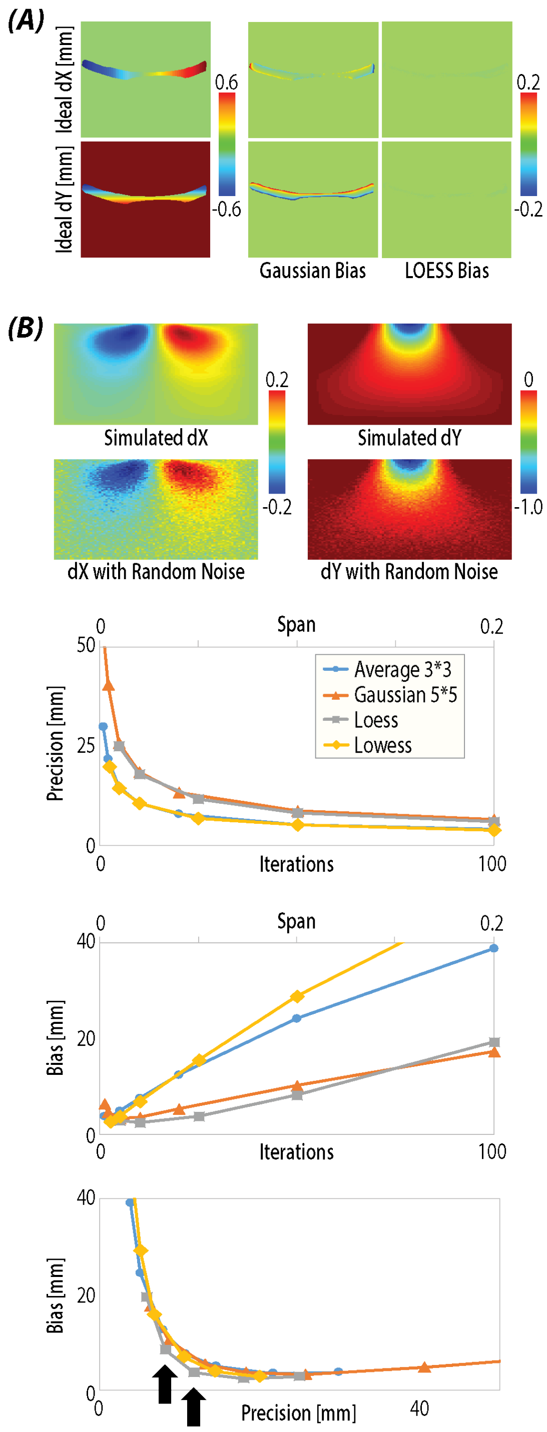

Supplemental Figure 1. Comparison showing local regression smoothing performs better in terms of preserving values at boundaries, with lower overall precision and bias. (A) Ideal gradient maps in horizontal and vertical direction were generated in a narrow mask and smoothed using Gaussian smoothing and local regression smoothing. While Gaussian smoothing exhibited bias values at boundaries, local regression smoothing did not. (B) Monte Carlo simulation was used to calculate precision and bias of both smoothing techniques. An indented rectangular 2D model was simulated in Abaqus and the displacement fields were used. Experimental level of random noise was generated 100 times and their smoothed results were compared to the input simulated displacements. It is important to note that the iteration is a Gaussian smoothing parameter and the span is the pixel considered in each local regression smoothing. In that last plot, precision and bias of both methods were combined.

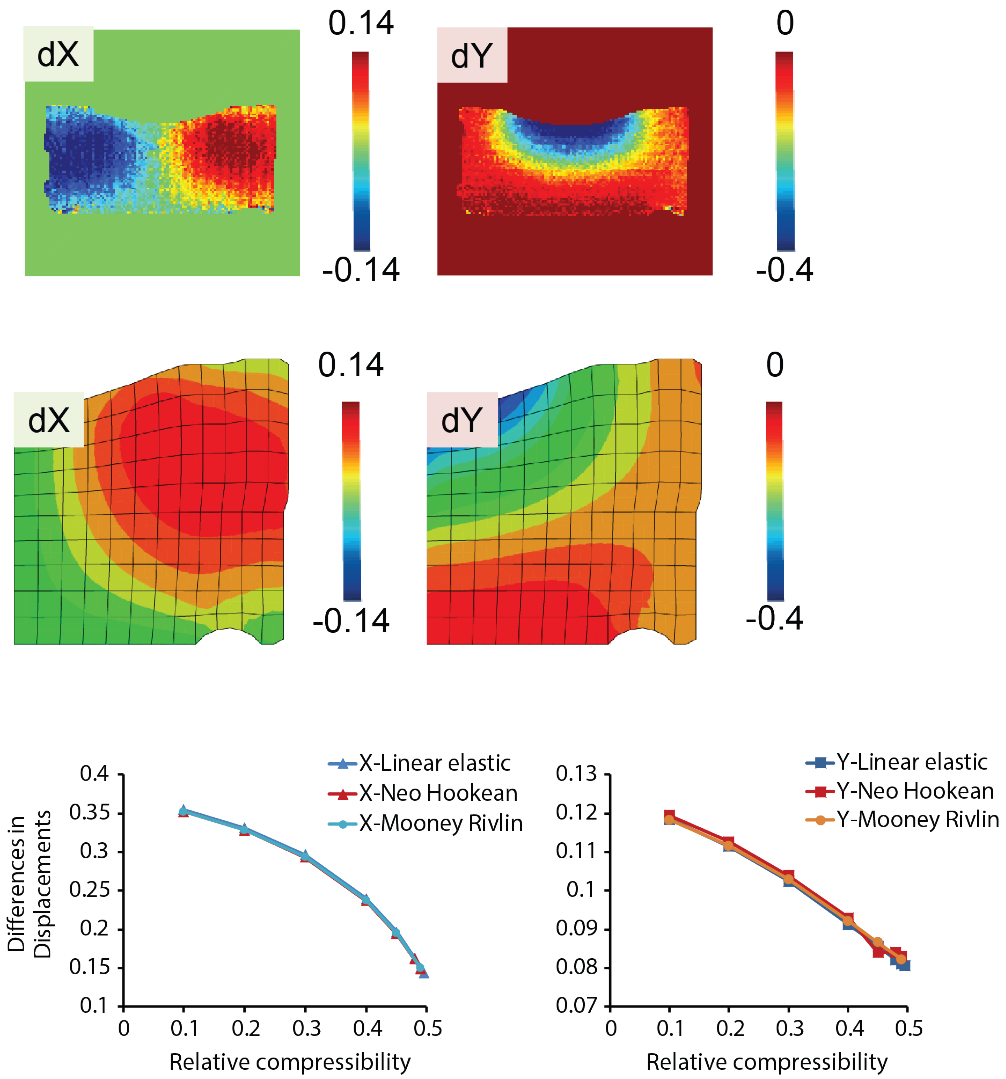

Supplemental Figure 2. Various constitutive models are all capable of inverse modeling within our finite deformation elastography framework. Displacement was measured in an unconfined loading test with a homogeneous, cylindrical hydrogel. An axisymmetric model was created in Abaqus with linear elastic and hyper elastic models and forward simulations was executed. With a relative compressibility (Poisson's ratio in linear elastic) of 0.5, showing nearly incompressible material, the difference of displacements between simulation and experiment demonstrated that the hydrogel behaved as an incompressible material and the effect of different material models was considered negligible.

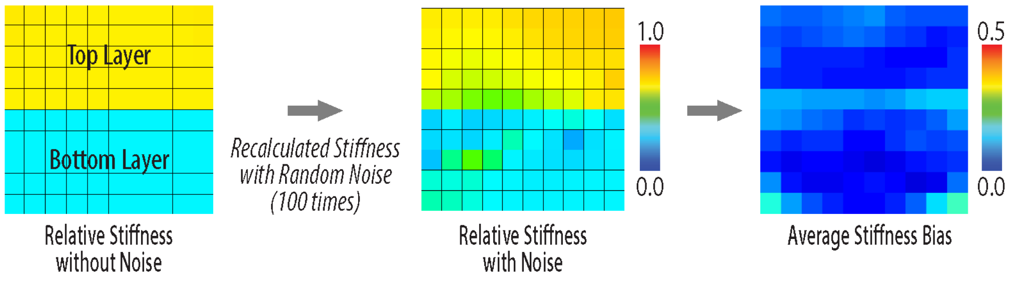

Supplemental Figure 3. Map of stiffness bias using Monte Carlo simulations. Bias was concentrated around the layer interface and near the fixed (bottom) edges.

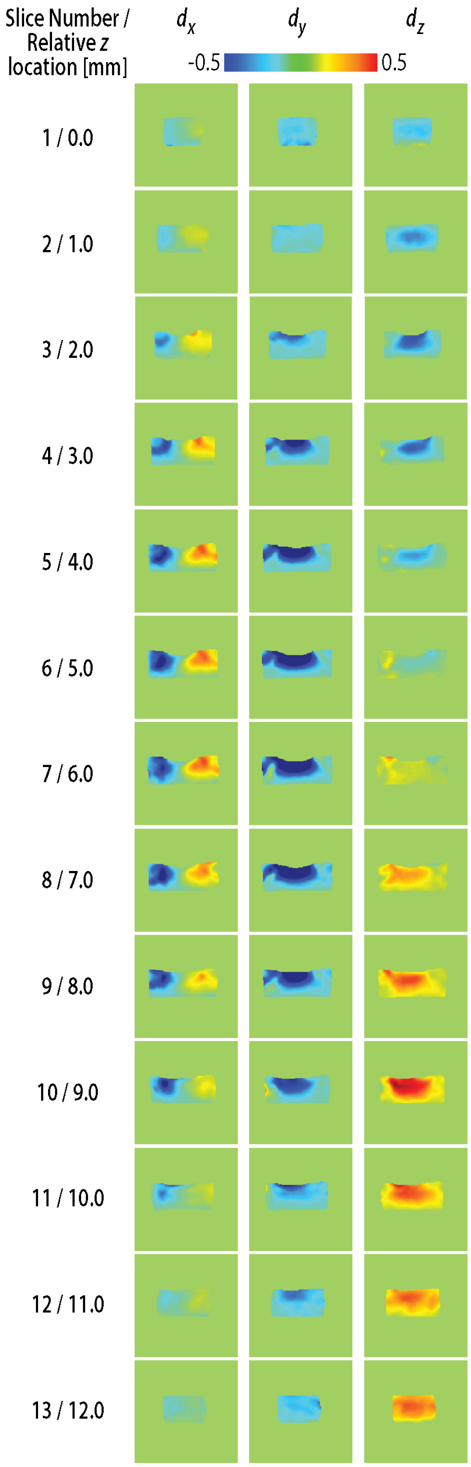

Supplemental Figure 4. Noninvasive mapping of *xyz* displacements measured in contiguous 13 slices to for 3D inverse modeling. Displacements were directly measured in volumetric (i.e. 2D multislice) images, enabling 3D stiffness mapping.
